# Electrochemical Broaching of Inconel 718 Turbine Mortises

**DOI:** 10.3390/ma18204732

**Published:** 2025-10-15

**Authors:** Shili Wang, Jianhua Lai, Shuanglu Duan, Jia Liu, Di Zhu

**Affiliations:** College of Mechanical & Electrical Engineering, Nanjing University of Aeronautics & Astronautics, 29 Yu Dao Jie Street, Nanjing 210016, Chinadzhu@nuaa.edu.cn (D.Z.)

**Keywords:** turbine mortise, electrochemical broaching, dissolution mechanism, experiment

## Abstract

The turbine mortise is a critical structural feature of turbine disks, and its manufacturing quality directly determines the performance and service life of aircraft engines. With the increasing application of advanced nickel-based superalloys, severe tool wear in conventional mechanical broaching of turbine mortises has emerged as a key limitation, substantially elevating production costs. Electrochemical broaching (ECB), which removes material through anodic dissolution reactions, eliminates tool wear and thus offers low cost and efficiency advantages, making it a promising method for turbine mortise fabrication. In this study, COMSOL Multiphysics 6.2 was employed to simulate the multiphysics field comprising the electric field, flow field, temperature field, bubble ratio, and dynamic mesh and elucidate the evolution of the electric field during the ECB process. ECB experiments of specimens on Inconel 718 were conducted under different feed speeds. On this basis, optimal processing parameters were identified. The results of the mid-position ECB experiments revealed five distinct dissolution states: pre-processing, pre-transition, stable dissolution, post-transition, and post-processing stages. A material dissolution mechanism model for the ECB process was established. Finally, fir-tree turbine mortises were successfully manufactured on Inconel 718 using a self-developed specialized electrochemical machining system at a feed speed of 70 mm/min. The mortise profile demonstrated dimensional deviations of (+16 to −21) μm, with working surface variations maintained within ±5 μm. The machined surfaces exhibited uniform and dense morphology with a surface roughness of Ra 0.275 μm. Three sets of mortise specimens processed under identical parameters showed excellent consistency, presenting a maximum deviation in profile removal thickness of +4.1 μm. The tool cathode was repeatedly reused without any detectable wear.

## 1. Introduction

The turbine mortise is a critical structural feature of turbine disks, and its manufacturing quality directly determines the performance and service life of aircraft engines. However, the fabrication of turbine mortises poses considerable challenges due to their complex geometry, their internal location within the turbine disk, the poor machinability of the materials involved, and stringent accuracy requirements. Among the key features of turbine mortises are narrow manufacturing tolerances and high surface quality. Typical mortise widths range from 20 to 40 mm, with shape accuracy maintained between 5 and 25 μm and surface roughness between 0.8 and 1.25 μm [1].

At present, mechanical broaching remains the primary manufacturing method for such mortises [2,3,4,5,6,7]. Nevertheless, with the growing use of advanced difficult-to-cut materials such as powder metallurgy superalloys, severe tool wear during broaching has become a major concern, significantly increasing production costs [8,9,10,11].

To overcome the limitations of mechanical broaching, researchers have explored alternative machining methods such as milling, grinding, and Wire electrical discharge machining (WEDM). Klocke et al. investigated a combined milling-broaching strategy and demonstrated that optimizing the process parameters and their proportions could significantly extend tool life [12]. WEDM has also been applied to turbine mortise fabrication, where optimized discharge parameters for roughing, finishing, and surface dressing were proposed [13,14,15,16]. Aspinwall et al. employed single-layer/electroplated cubic boron nitride and diamond grinding wheels for mortise machining, analyzing the effect of cutting speed on tool wear and surface integrity [17]. Shi et al. examined the influence of work speed, depth of cut, and up-/down-grinding strategies on net grinding power and surface burn [18]. Li et al. studied the effects of electroplated CBN form wheels and localized single-sided form grinding on profile accuracy, surface quality, and wheel wear [19]. Ding et al. further designed a cup-shaped CBN wheel, developed a grinding force model, and investigated the effects of grinding parameters and slot geometry on grinding forces [20,21]. Farooq et al. proposed a novel flushing mechanism and analyzed the influence of process parameters on the geometric error, spark gap formation, and arithmetic surface roughness of the nickel-based alloy GH4169 [22]. Buk et al. investigated the effects of peak current and feed parameters during WEDM finishing on the surface properties of GH4169 [23]. They further proposed a parameter control method capable of achieving the desired shape accuracy, surface roughness, and surface layer conditions [24]. Similarly, Sun et al. developed predictive mathematical models for the relationship between surface roughness, kerf width, and discharge parameters, and provided predictions and corrections for the offset of each cut in multi-pass cutting [25]. However, in the case of form-tool milling or grinding, the small tool diameter and low cutting speed result in severe tool wear when machining difficult-to-cut materials such as powder metallurgy superalloys. For WEDM, the inherent material removal mechanism inevitably leads to the formation of a recast layer and other surface integrity issues. Therefore, the development of efficient and cost-effective machining technologies for turbine mortises is both urgent and essential.

Electrochemical broaching (ECB) is a nonconventional machining technique analogous in form to mechanical broaching, which relies on the principle of anodic dissolution for material removal. This method offers significant advantages, such as being unaffected by workpiece hardness and toughness, absence of tool wear, high efficiency, and superior surface quality. These attributes make ECB particularly cost-effective for machining difficult-to-cut materials [26,27,28]. Recently, advances in machining accuracy have established ECB as a promising technology for the efficient and economical fabrication of turbine mortises. However, research on the material dissolution behavior of nickel-based superalloys during ECB remains unexplored. When the cathode tool passes over the workpiece surface, the local current density exhibits a dynamic variation characterized by successive stages of increase, high-level maintenance, and subsequent decrease. This non-steady-state electric field distribution leads to a dissolution behavior significantly different from traditional copy-type electrochemical machining. In particular, the decay phenomenon during the current decline phase induces persistent stray current corrosion, and its correlation with process parameters has not been thoroughly revealed in existing studies.

To elucidate this unique dissolution mechanism, COMSOL Multiphysics 6.2 was employed to simulate the multiphysics field comprising the electric field, flow field, temperature field, bubble ratio, and dynamic mesh, elucidate the evolution of the electric field during the ECB process, and predict the material removal amount. Through planar ECB experiments, the material removal amount and surface quality under varying feed speeds were analyzed. Building on this, intermediate position machining experiments were conducted. The evolution of surface morphology across the pre-processing, stable dissolution, and post-processing stages was systematically analyzed. Finally, experiments were conducted on fir-tree turbine mortises made of Inconel 718, validating the feasibility of the process.

## 2. Principle of ECB

**Figure 1:**
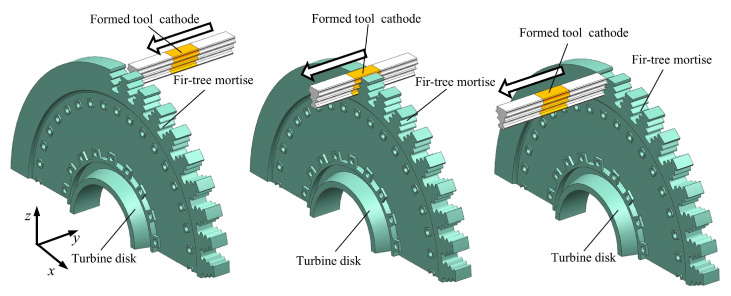
ECB schematic of turbine mortise.

A schematic diagram of the turbine mortise ECB method is shown in Figure 1 The formed cathode tool, whose cross-sectional profile gradually expands along the feed direction, is connected to the negative pole of the power supply, while the mortise-profiled workpiece blank with machining allowance is connected to the positive pole. An electrolyte with a certain pressure and flow speed passes through the machining gap in a direction parallel to the feed motion. Once the power supply is switched on, intensive electrochemical reactions occur within the machining gap: the anode material of the workpiece undergoes continuous dissolution, while hydrogen bubbles are simultaneously generated on the cathode surface. As the tool electrode with a gradually expanding cross-section rapidly advances across the workpiece surface, the full contour of the mortise structure is progressively shaped, ultimately achieving the final formation of the turbine mortise.

**Figure 2:**
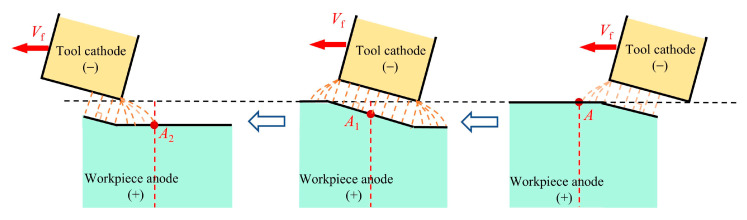
ECB machining process.

Taking a cross-section of the anode workpiece perpendicular to the feed direction of the tool as a reference, the ECB process of the tool cathode passing through the workpiece anode is illustrated in Figure 2. According to the relative position between the tool cathode and the workpiece anode, the process can be divided into three stages: pre-processing, in-processing, and post-processing. In the pre-processing stage, the tool cathode rapidly approaches the workpiece anode, and as the inter-electrode gap narrows, the current density on the workpiece anode contour increases sharply. When the front end of the tool cathode passes the workpiece, the process enters the in-processing stage, during which the current density on the workpiece contour reaches its peak, leading to accelerated dissolution of the workpiece material. The horizontal feed of the inclined tool cathode can be equivalently regarded as a downward feed, maintaining a small gap and high current density between the tool and the workpiece. After the rear end of the tool cathode passes the workpiece, the process enters the post-processing stage. At this point, the gap between the tool cathode and workpiece anode rapidly widens, the current density drops significantly., and stray currents at the edges of the machining gap cause secondary corrosion of the machined surface until the process is completed.

Among the three stages of the ECB process, the in-processing and post-processing stages directly determine the surface quality of ECB. In particular, during the post-processing stage, as the tool electrode gradually moves away from the reference section of the workpiece at a given feed speed, the surface current density of the workpiece may require several seconds or even tens of seconds to approach zero. The waveform of current density variation on the workpiece anode surface during the post-processing stage has a significant influence on the material dissolution behavior. Therefore, to clarify the dissolution mechanism under ECB conditions, it is first necessary to understand the evolution characteristics of the electric field during the process.

## 3. Electric Field Evolution in ECB

To reveal and understand the evolution law of the electric field during ECB, a dynamic multiphysics coupling simulation of the ECB process was conducted by COMSOL Multiphysics 6.2. The following assumptions were made in the simulation [29,30,31]:

(1) Ohm’s law is valid on the cathode surface, the anode surface, and throughout the machining zone;

(2) The influence of anode dissolution products on the conductivity within the machining gap is neglected, while the effects of hydrogen bubbles generated on the cathode and the Joule heating–induced temperature rise on conductivity are considered;

(3) The material dissolves uniformly, and the current efficiency (η) of the anodic dissolution remains constant throughout the entire process;

(4) The tool cathode and workpiece anode boundaries are treated as equipotential surfaces, and the effect of polarization potential is neglected.

**Figure 3:**
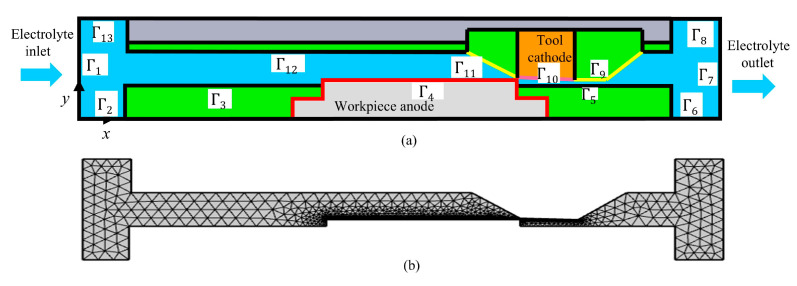
Dynamic simulation model and mesh generation of ECB. (**a**) Dynamic simulation. (**b**) Mesh generation.

Figure 3 illustrates the geometric model used for the dynamic simulation of ECB. In the simulation, the length of the tool cathode is 6 mm, and its machining surface is inclined, with a height difference of 0.2 mm between the front and rear end faces. The vertical distance from the left-end face of the tool cathode to the anode surface of the workpiece is also 0.2 mm. In the model, Γ4 represents the anode surface boundary of the workpiece, and Γ10 denotes the cathode boundary of the tool. Γ1 is defined as the electrolyte inlet boundary, and Γ7 as the electrolyte outlet boundary, while the remaining surfaces are treated as solid walls corresponding to the insulation of fixtures and tooling.

In the machining zone, the potential distribution can be approximated by the Laplace equation:

(1) $$\vec{v_{n}}=\eta \omega \vec{i_{n}},$$

where *η* is the current efficiency and *ω* is the volumetric electrochemical equivalent of the workpiece material.

During machining, a hydrogen reduction reaction occurs at the cathode surface. Based on Faraday’s law, the amount of hydrogen generated on the cathode surface satisfies the following:

(2) $$N_{H_{2}}=\frac{M_{H_{2}}}{2F},$$

where $N_{H_{2}}$ is the molar mass of hydrogen, *F* is Faraday’s constant, and *i* is the local current density on the cathode surface.

In the process, Joule heating generated by the current flowing through the electrolyte is the primary source of temperature rise within the machining gap, expressed as follows:

(3) $$P=\frac{I^{2}}{\kappa },$$

where *I* is the current and *κ* is the electrolyte conductivity. The relationship among electrolyte conductivity, void fraction of hydrogen bubbles, and temperature is given by the following:

(4) $$\kappa =\kappa_{0}{\left(1-\beta \right)}^{m}\left(1+\lambda \left(T-T_{0}\right)\right),$$

where $\kappa_{0}$ is the initial conductivity of the electrolyte, *β* is the void fraction of gas bubbles in the electrolyte, *T* is the electrolyte temperature, $T_{0}$ is the initial electrolyte temperature, *m* is the correction coefficient of bubble fraction on conductivity, and *λ* is the temperature-dependent gradient.

In the simulation, the entire machining process is discretized into a series of very short time intervals Δ*t*. Within each, the electric field and electrolyte flow field distributions in the machining gap are first calculated based on the boundaries of the tool cathode and workpiece anode. Then, according to the electric field distribution on the cathode and anode surfaces, the generation of hydrogen bubbles and the Joule heating induced temperature rise within the gap are solved, from which the conductivity distribution of the electrolyte along the flow direction is obtained. Subsequently, the amount of material removed from the anode boundary during Δ*t* is determined, thereby forming a new anode boundary. The material removal depth within Δ*t* can be expressed as follows:

(5) $$\Delta d\;=\;\nu_{n}\;\times \;\Delta t$$

Meanwhile, the contour profile of the tool cathode is translated by a certain distance according to the specified feed speed and feed direction over the duration of Δ*t*, thus forming a new cathode boundary. By repeating the above procedure until the end of machining, the material removal process in ECB can be simulated, and the dynamic evolution of the machining current density on a specific cross-section of the workpiece anode can be obtained. The multiphysics coupling simulation parameters are listed in Table 1.

**Figure 4:**
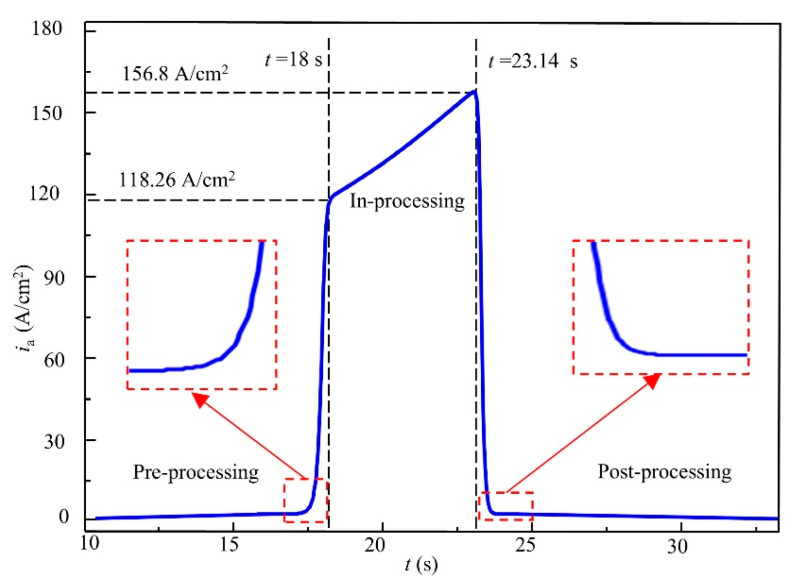
Current density variation at point A on anode surface.

Figure 4 shows the variation in current density on the anode surface at reference point A during the approach, passage, and departure of the tool cathode. The tool cathode feeds from left to right. When the simulation time reaches 10 s, the left end face of the cathode is approximately 9.3 mm away from point A, and the current density at point A is about 0.16 A/cm^2^. As the cathode continues to approach point A, the current density at point A exhibits a gradual increase. When the simulation time reaches 16 s, with the left end face of the cathode approximately 2 mm from point A, the current density begins to rise rapidly. At 18 s, when the left end face of the cathode is located directly above point A, the current density reaches 118.26 A/cm^2^. The simulated current density evolution in the pre-processing stage shows agreement with the previous analysis of the principle of ECB.

When the tool cathode passes the reference point and the right end face of the cathode is located above point A, the process enters the in-processing stage, corresponding to the simulation period from 18 s to 23.14 s. During this stage, the current density at point A continues to increase as machining progresses, reaching a peak value of 156.8 A/cm^2^ when the right end face of the cathode is directly above point A. The continuous rise in current density at point A during the in-processing stage is mainly attributed to the inclined surface of the ECB cathode, which introduces a vertical feed component during the horizontal feed motion. This vertical component exceeds the downward dissolution rate of point A, thereby reducing the local machining gap and causing a sustained increase in machining current. This finding indicates that even during the in-processing stage, ECB does not exhibit a steady-state machining phase, which is in sharp contrast to conventional copy-type electrochemical machining.

After the right end face of the tool cathode moves away from the reference point, the ECB process enters the post-processing stage, corresponding to the simulation time after 23.14 s. As shown in Figure 4, within just 1 s, the current density at point A decreases sharply from 156.8 A/cm^2^ to 10 A/cm^2^. Thereafter, the speed of decline slows significantly, and even after 8 s, the current density on the anode surface remains at 0.8 A/cm^2^. The long-lasting exposure of the machined surface to stray currents at the gap edges results in secondary corrosion, which has a significant impact on the material dissolution behavior.

**Figure 5:**
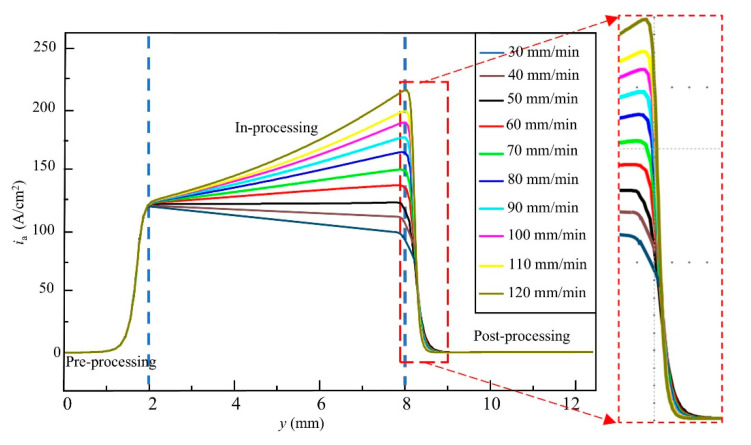
Current density variation under different feed speeds.

**Figure 6:**
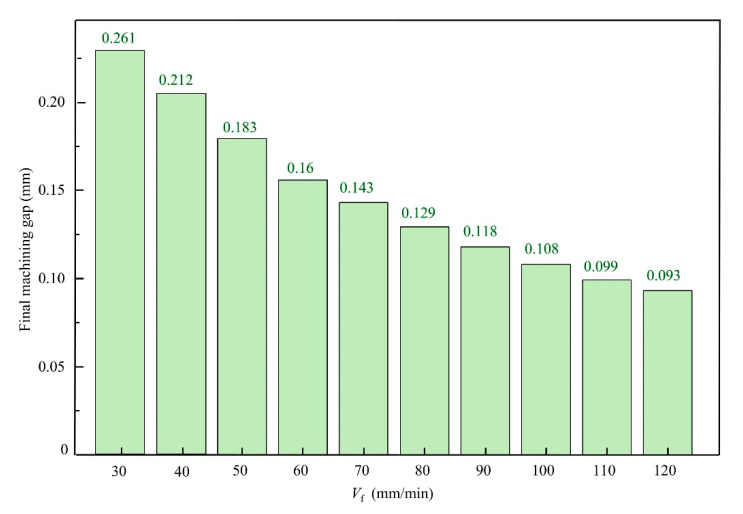
Final machining gap versus feed speed from simulations.

To further investigate the phenomenon of continuous current density, increase during the in-processing stage, ECB simulations were conducted under different feed speeds. The feed speeds ranged from 30 mm/min to 120 mm/min with an increment step of 10 mm/min, resulting in a total of nine simulation cases. Since the duration of the in-processing stage varies with feed speed, the simulation results were normalized by using the distance between the front end of the tool cathode and point A as the horizontal axis. The current density evolution curves under different feed speeds are shown in Figure 5, while the final machining gaps (removal thickness) obtained from the simulations are presented in Figure 6. The results indicate that when the feed speed is 30 mm/min, the current density on the anode surface shows a decreasing trend, with the current density at the end of the in-processing stage being lower than that at the beginning by 105 A/cm^2^. The final machining gap in this case is 0.261 mm, which is larger than the initial gap of 0.2 mm. As the feed speed increases, the current density on the anode surface gradually exhibits an increasing trend. At a feed speed of 120 mm/min, the peak current density reaches approximately 216 A/cm^2^, and the final machining gap is reduced to 0.102 mm. These simulation results confirm the conclusions drawn from the previous current density waveform analysis.

## 4. Dissolution Behavior in ECB

This study employs planar ECB as an efficient experimental method to investigate the material dissolution behavior and surface quality control strategies for complex structures, particularly the turbine mortise. The studies provided important theoretical and technical support for high-quality manufacturing of these specialized geometries.

**Figure 7:**
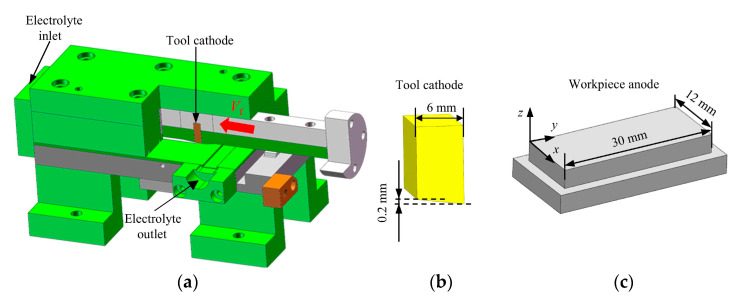
Experimental setup for ECB machining of specimen. (**a**) Fixture. (**b**) Tool cathode. (**c**) Workpiece anode.

To investigate the material dissolution behavior under ECB, planar specimen experiments were carried out under different feed speeds. The experimental setup is shown in Figure 7. The length of the tool cathode was 6 mm, with an angle of 1.91° (the height difference across the tool cathode’s end faces corresponds is 0.2 mm), and the tool material was tungsten–copper alloy. The workpiece anode was a flat plate with dimensions of 30 mm × 12 mm, fabricated by WEDM, and made of Inconel 718. The chemical composition of the alloy is listed in Table 2. To ensure a stable electrolyte flow field in the machining gap during ECB, a fixture was designed to guide and seal the electrolyte, which was manufactured from insulating non-metallic materials.

**Figure 8:**
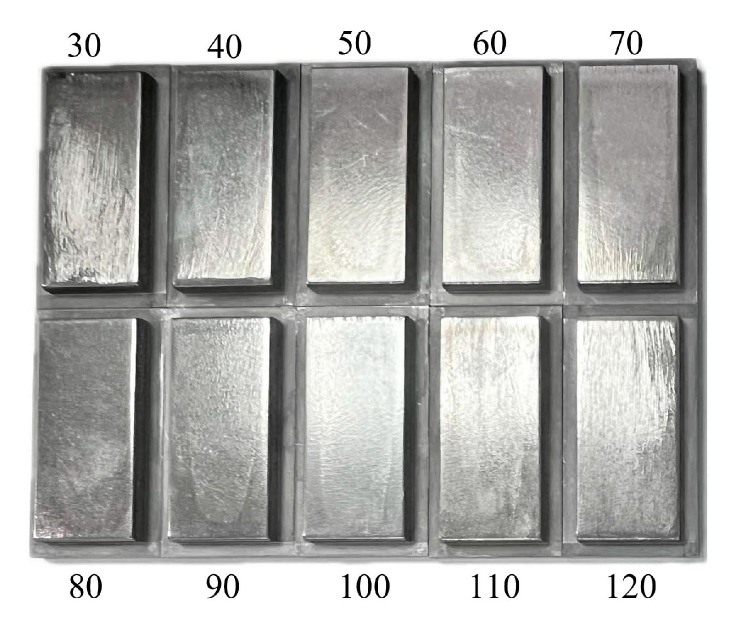
Specimens at different feed speeds (mm/min).

**Figure 9:**
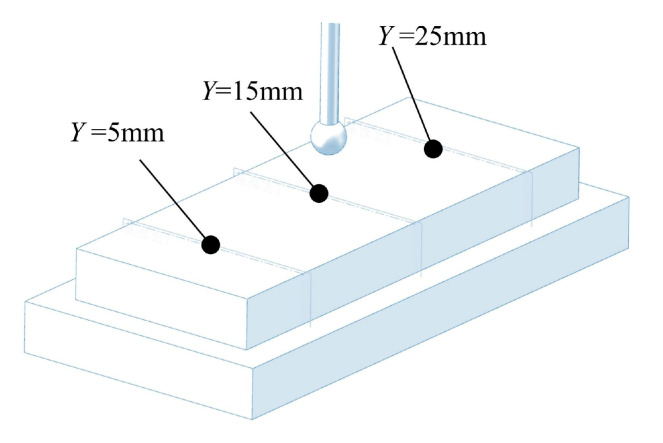
Planar measurement by CMM.

**Figure 10:**
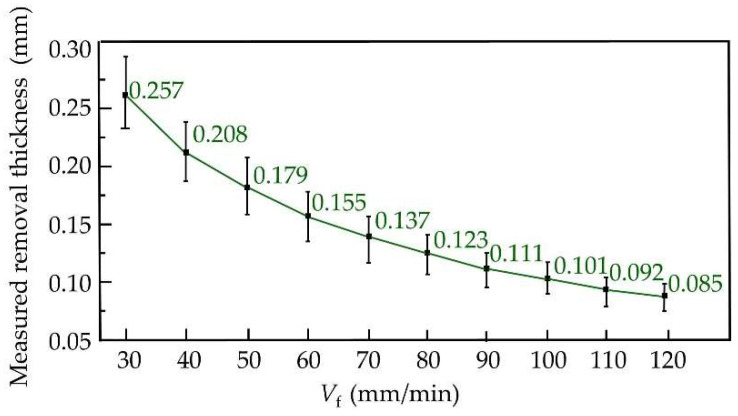
Measured removal thickness at different feed speeds.

The experimental parameters of ECB were consistent with those used in the simulation, as listed in Table 1. A total of nine experiments were conducted with feed speeds ranging from 30 mm/min to 120 mm/min in increments of 10 mm/min. The machined specimens are shown in Figure 8. The removal thickness at different locations on the workpiece surface was measured using a Coordinate Measuring Machine (CMM, Hexagon, Stockholm, Sweden). The measurement strategy is illustrated schematically in Figure 9, and the corresponding measurement results are shown in Figure 10.

**Figure 11:**
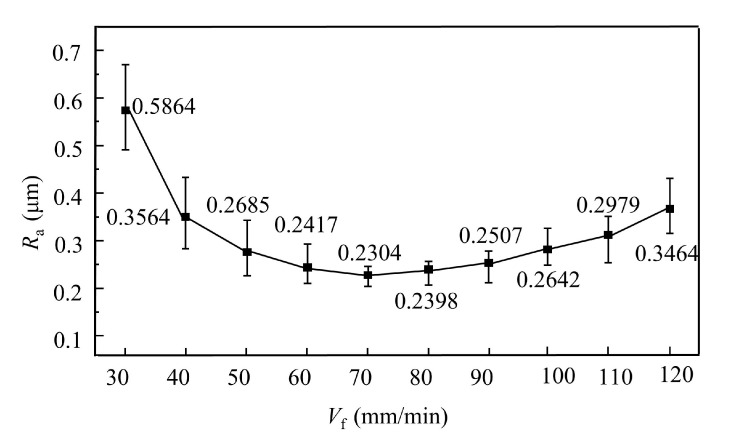
Average surface roughness at different feed speeds.

The surface topography and roughness of each specimen were measured using a Super-Depth-of-Field Microscope (SDM, KEYENCE, Osaka, Japan). For each specimen, five positions were selected, and three measurements were performed at each position. The resulting surface roughness results of the ECB processed specimens at different feed speeds are presented in Figure 11.

**Figure 12:**
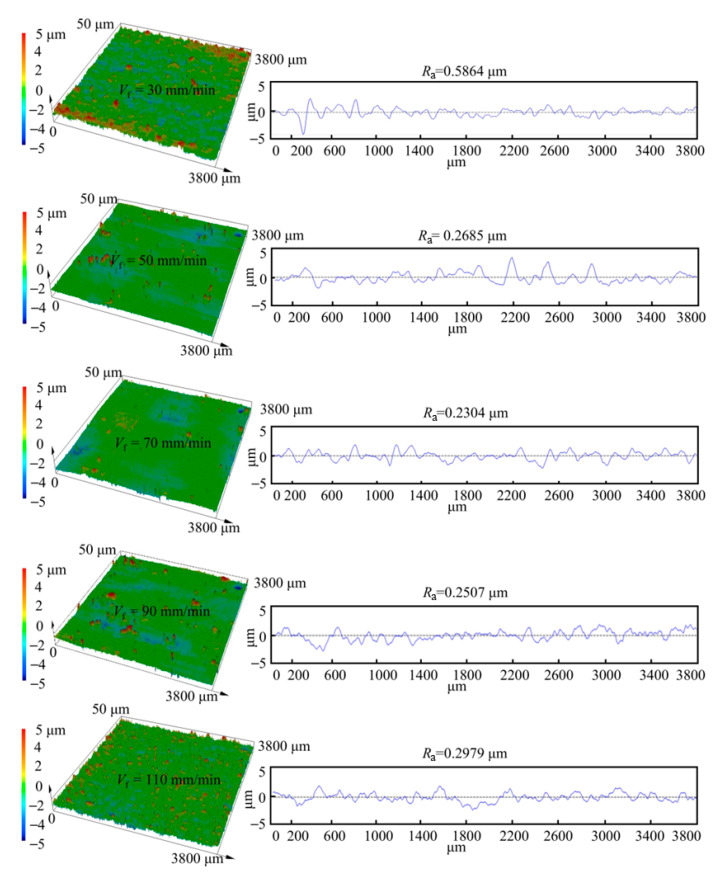
3D surface morphology and roughness results at different feed speeds.

The experimental results show that at a feed speed of 30 mm/min, the surface roughness was 0.5864 μm. As the feed speed increased, the surface roughness decreased, reaching 0.2304 μm at 70 mm/min. When the feed speed was further increased, however, the surface roughness rose again, reaching 0.2979 μm at 110 mm/min. With increasing feed speed, the machining gap gradually changes from an expanding state to a shrinking state. When the machining gap expands, the current density on the machined surface decreases, leading to reduced surface quality. Conversely, when the machining gap shrinks, the surface current density increases, but due to the excessively small gap, electrolytic products cannot be removed in time, and the accumulation of temperature and gas bubbles results in surface quality deterioration. The surface morphology corresponding to the surface roughness at different feed speeds, as observed by using the SDM, is shown in Figure 12.

To further analyze the surface evolution behavior of the material during the pre-processing, in-processing, and post-processing stages of ECB, a mid-position ECB experiment was carried out. In this experiment, the tool cathode was continuously fed until its left end reached the middle position of the workpiece, at which point the process was stopped. Based on the relative position between the tool cathode and the workpiece anode, the entire surface evolution process corresponding to the pre-processing, in-processing, and post-processing stages could be observed on the specimen. The mid-position ECB experiment was conducted at a feed speed of 70 mm/min, and the experimental parameters were consistent with those used in the simulations.

**Figure 13:**
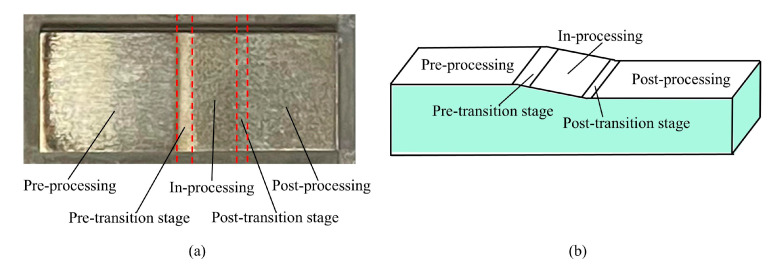
Specimen obtained from mid-position ECB machining. (**a**) Planar specimen. (**b**) Schematic diagram.

**Figure 14:**
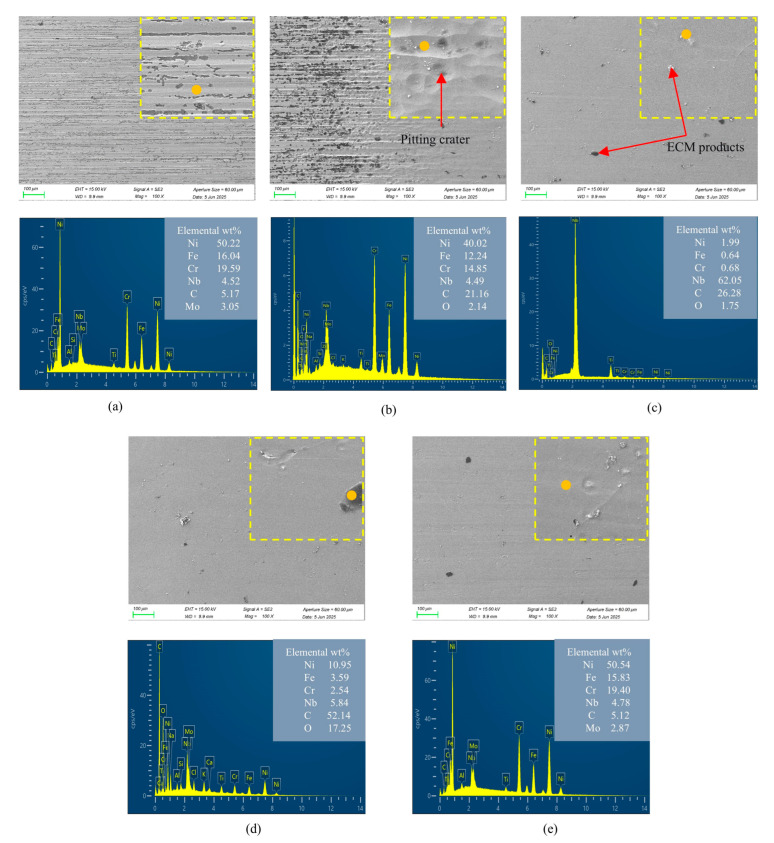
Dissolution morphology and elemental composition of Inconel 718. (**a**) Pre-processing stage. (**b**) Pre-transition stage. (**c**) In-processing stage. (**d**) Post-transition stage. (**e**) Post-processing stage.

The specimen of the mid-position ECB experiment was shown in Figure 13. The experimental observations indicate that within approximately 2 mm before and after the in-processing stage, there exists a distinct transition region in surface quality, here referred to as the pre-transition stage and post-transition stage. A scanning electron microscope with an energy-dispersive spectrometer (SEM-EDS, ZEISS, Oberkochen, Germany) was employed to analyze the surface morphology and elemental composition of each region, respectively. The surface evolution process obtained from these analyses is presented in Figure 14.

**Figure 15:**
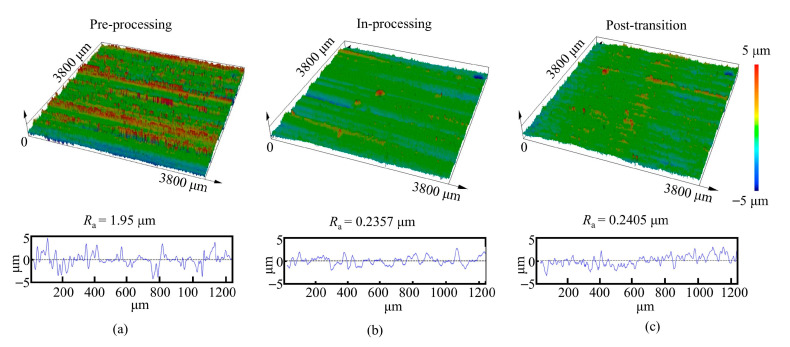
Three-dimensional morphology and surface roughness of Inconel 718 alloy. (**a**) Pre-processing stage. (**b**) In-processing stage. (**c**) Post-processing stage.

On the left side of the pre-transition stage, the material remains uncorroded, still exhibiting the original surface characteristics from WEDM, with pronounced ridges and grooves along the feed direction of the wire electrode. The pre-transition stage corresponds to the process in which these ridges and grooves are gradually corroded by the increasing current density. As the tool cathode continuously approaches, the current density increases, and the protruding ridges are dissolved first. When the machined surface enters the high current density region, the protrusions on the anode surface are completely dissolved, and no WEDM traces remain. Elemental analysis of the WEDM surface shows that nickel accounts for 50.22% and carbon for 5.17%. During electrochemical dissolution, the oxygen content increases due to the precipitation of reaction products on the anode surface. The white precipitates are mainly Nb phases, while the black deposits are rich in carbon, identified as MC-type carbides. From the in-processing to the post-processing stage, another transition region of approximately 2 mm in width is observed. The surface morphology in this region is similar to that in the in-processing stage, though the surface quality is slightly inferior. As the machined surface gradually moves away from the tool cathode, the surface morphology and quality of the anode tend to stabilize. The evolution of surface roughness across the pre-processing, in-processing, and post-processing stages is shown in Figure 15.

**Figure 16:**
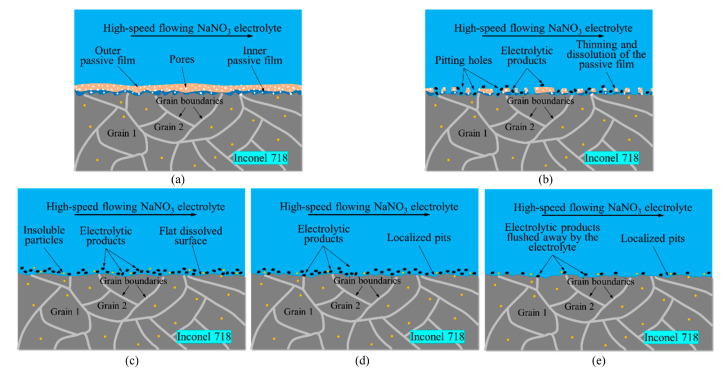
ECB dissolution model of Inconel 718 alloy in 20wt.% NaNO_3_ solution. (**a**) Pre-processing stage. (**b**) Pre-transition stage. (**c**) In-processing stage. (**d**) Post-transition stage. (**e**) Post-processing stage.

Based on the above analysis, the ECB dissolution process of the nickel-based alloy Inconel 718 can be divided into five typical stages, and the corresponding qualitative dissolution model is illustrated in Figure 16. In the pre-processing stage (Figure 16a), spark pits generated during wire WEDM are stacked on the surface, forming pronounced ridges and grooves. Meanwhile, the alloy surface is covered by a stable and compact oxide film composed of a dense and uniform inner layer and a porous outer layer. In the pre-transition stage (Figure 16b), as the electric field intensity gradually increases, electrochemical reactions cause the initial passive film to thin and eventually break down. Due to the higher electrochemical activity of ridge areas, localized breakdown of the passive film preferentially occurs at these sites, exposing the substrate to the electrolyte. This rapidly initiates electrochemical pitting and produces a roughened surface. In the stable dissolution stage (Figure 16c), the continuous increase in current density promotes the growth and coalescence of numerous pits. Both grain boundaries and grain interiors undergo electrochemical dissolution, expanding the corroded area. As a result, the overall surface morphology becomes relatively flat. In the post-transition stage (Figure 16d), the sharp decrease in current density weakens the electric field, and the dissolution mode shifts from uniform corrosion to localized pitting. The surface exhibits sporadically distributed electrolytic products and insoluble particles. Finally, in the post-processing stage (Figure 16e), as the tool cathode gradually moves away from the workpiece surface, the dissolution in the machining zone approaches termination. The high-speed electrolyte flow effectively removes the reaction products, bubbles, and heat, leaving a surface that is relatively clean and smooth, with only scattered residual pits.

## 5. ECB of Turbine Mortise

**Figure 17:**
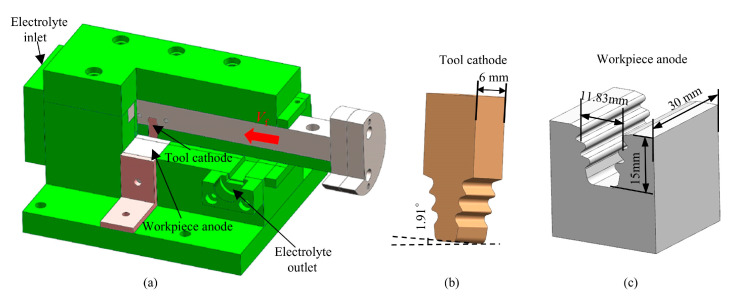
Experimental setup used in turbine mortise ECB machining. (**a**) Fixture. (**b**) Tool cathode. (**c**) Workpiece anode.

The turbine mortise specimens machined by ECB are shown in Figure 17. Each mortise had a length of 30 mm, a maximum width of 11.83 mm, and a depth of 15 mm. The mortise contour consisted of smoothly connected circular arcs with different radii and straight segments. The workpiece material was Inconel 718. The tool cathode was designed with a fir-tree shape and an inclined angle, with a length of 6 mm and the angle of 1.91° (the height difference across the tool cathode’s end faces corresponds is 0.2 mm). and the tool material was tungsten–copper alloy. The dimensions of the large end of the cathode matched the blank size of the mortise specimen. Both the mortise specimens and tool cathodes were fabricated by wire WEDM. Flow-guiding structures were designed at both the front and rear of the tool cathode. The front section adopted an expanding fir-tree-shaped guide to introduce the electrolyte into the machining zone rapidly, uniformly, and stably, while the narrowing rear guide section provided back pressure and simultaneously directed the electrolyte to promptly remove reaction products and heat. The electrolyte inlet direction was opposite to the feed direction of the cathode. The ECB experiments on turbine mortise specimens were conducted using the same machine tool, high-power DC supply, and electrolyte circulation system as in the mid-position planar ECB experiments. The applied voltage was 20 V, and the electrolyte was 20 wt.% NaNO_3_ solution. The inlet and outlet pressures of the electrolyte were 1.3 MPa and 0.2 MPa, respectively, with the electrolyte temperature maintained at 30 ± 0.5 °C. The initial machining gap was set to 0.2 mm. The feed speed *V*_f_ of ECB was 70 mm/min. The experimental setup is shown in Figure 17.

**Figure 18:**
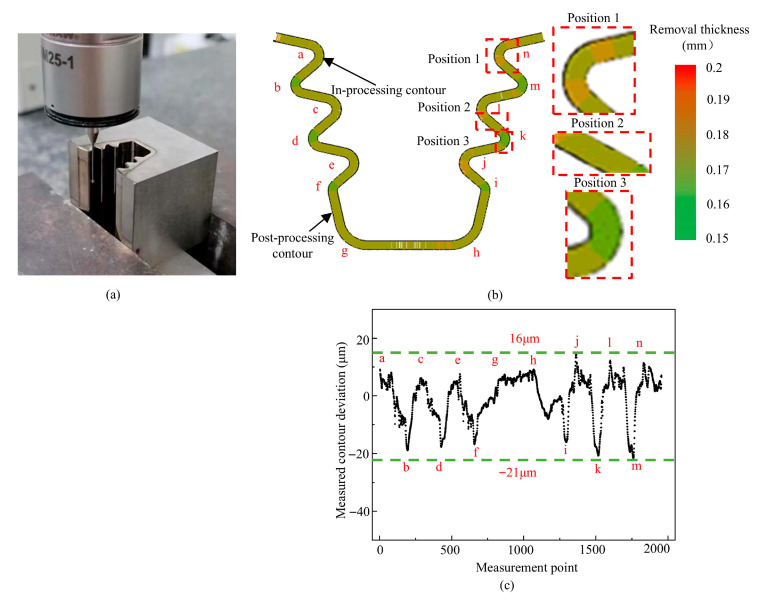
Material removal distribution of the fir-tree turbine mortise by ECB. (**a**) CMM measurement. (**b**) Removal thickness distribution. (**c**) Profile deviation along the arc length.

The contour of the fir-tree turbine mortise was measured using CMM, shown in Figure 18. The material removal thickness and the deviation of the complete contour are shown in Figure 18b, ”a–n” are the sequential designations for the arc segments in the fir-tree turbine mortise contour. At position 1, the removal thickness was 0.158 mm; at position 2, it was 0.146 mm; and at position 3, it was 0.127 mm. For each specimen, five positions were selected, and three measurements were performed at each position. The measured contour deviation of the fir-tree turbine mortise ranged from +16 μm to –21 μm.

**Figure 19:**
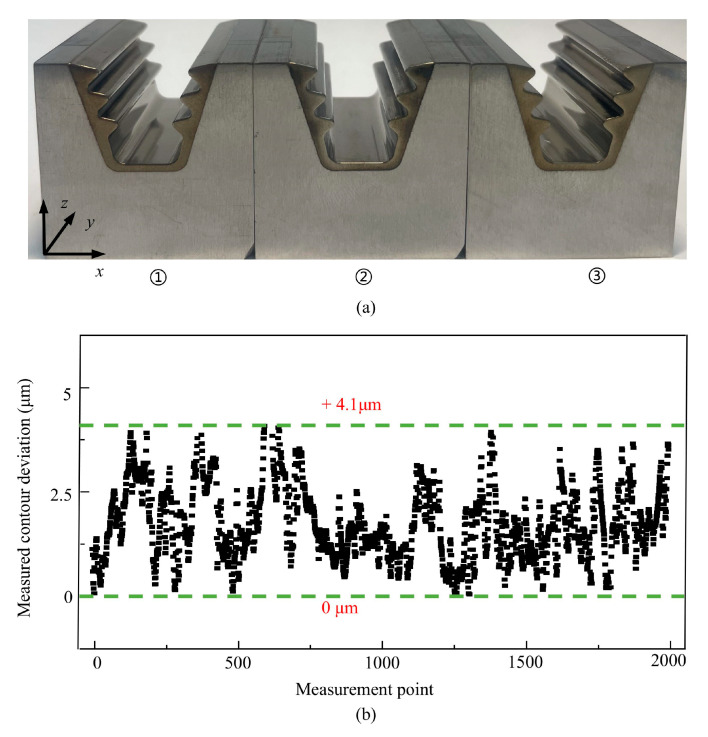
Three machined specimens and Profile repeatability. (**a**) Three sets of fir-tree turbine mortise specimens. (**b**) Repeatability of three specimens.

The three sets of the fir-tree mortise specimens processed under identical technological parameters all demonstrated excellent consistency. The maximum deviation in contour removal thickness was +4.1 μm, while the height deviation was controlled within 3 μm, as shown in Figure 19.

**Figure 20:**
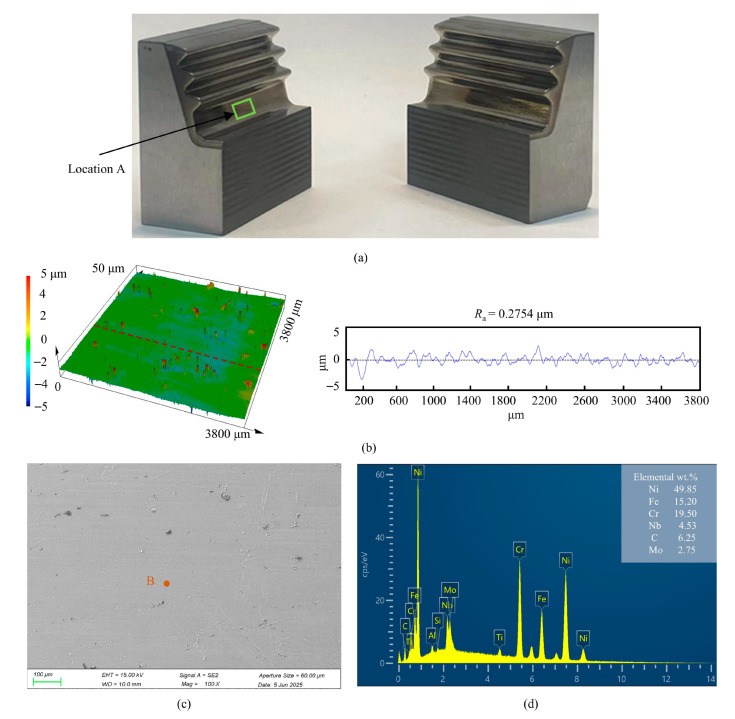
Surface roughness and elemental composition of the fir-tree turbine mortise. (**a**) Sectioned specimen. (**b**) 3D morphology and surface roughness. (**c**) Dissolution morphology. (**d**) Elemental composition of dot B.

The fir-tree mortise specimen was sectioned longitudinally for analysis. As shown in Figure 20b, the surface roughness at location A was measured to be Ra 0.2754 μm, and its corresponding surface morphology was characterized. Furthermore, the microscopic morphology and elemental distribution at the same location are presented in Figure 20c. The surface quality of the fir-tree mortise is essentially consistent with that of the planar sample mentioned above by ECB, and the mortise edges exhibited smooth transitions without sharp corners or burrs.

## 6. Conclusions

This study investigated the ECB process of Inconel 718 turbine mortises through multiphysics simulations, planar specimen experiments, and fir-tree turbine mortise machining experiments. The main conclusions can be summarized as follows:

(1) A dynamic multiphysics coupling model of ECB was established to analyze the evolution of the electric field and current density within the machining gap. The simulation results revealed that the current density at the anode surface undergoes three distinct stages (pre-processing, in-processing, and post-processing), with a continuous rise during the in-processing stage, indicating that ECB does not exhibit a steady-state machining phase, unlike conventional copy-type electrochemical machining.

(2) ECB experiments of planar specimens under different feed speeds showed that feed speed significantly influences surface roughness and machining gap. The optimal surface quality was achieved at a feed speed of 70 mm/min, with a surface roughness of 0.2304 μm. Excessively low or high feed speeds led to surface deterioration due to insufficient dissolution or the accumulation of bubbles and heat, respectively.

(3) The mid-position ECB experiments revealed complete surface evolution across pre-processing, in-processing, and post-processing stages. Based on the super-depth optical microscope and an energy dispersive spectrometer analysis, a five-stage dissolution model of Inconel 718 in ECB was proposed, consisting of the pre-processing, pre-transition, stable dissolution, post-transition, and post-processing stages. This model clarifies the dynamic dissolution mechanisms of nickel-based superalloys under ECB conditions.

(4) The fir-tree turbine mortises were successfully broached in Inconel 718 using ECB. The processed specimens exhibited a contour deviation ranged from +16 μm to –21 μm. and a bottom surface roughness of Ra 0.2754 μm, with smooth edge transitions and no burrs or sharp corners. These results confirm the feasibility of ECB for turbine mortise fabrication, offering significant advantages in precision, efficiency, and surface integrity over conventional machining methods.

## Figures and Tables

**Table 1:** Simulation parameters for the ECB multiphysics coupling machining process.

Parameter (Unit)	Value
Machining voltage *U* (V)	20
Workpiece anode material	Inconel-718
Electrolyte	20wt.% N_a_NO_3_
Initial machining gap in ECB Δ_0_ (mm)	0.2
Feed speed of cathode tool ${\;V}_{f}$ (mm/min)	70
Electrolyte inlet pressure *P*_in_ (MPa)	1
Electrolyte outlet pressure *P*_out_ (MPa)	0.3
Initial electrolyte temperature *T*_0_ (℃)	30 ± 0.5
Electrolyte Conductivity κ (S/m)	14.3
Tool Cathode Material	tungsten–copper alloy
The actual electrochemical volume equivalent *ηω-j* (mm^3^·A^−1^·min^−1^)	1.42

**Table 2:** Chemical composition of Inconel 718 alloy (wt.%).

Element	C	Fe	Cr	Mo	Ti	Ni	Nb	Al
Content	0.08	18.7	19.1	3.01	0.91	BaL.	4.86	0.52

## Data Availability

The original contributions presented in this study are included in the article. Further inquiries can be directed to the corresponding author.

## Nomenclature

*η* *ω*	The actual electrochemical volume equivalent
*ν*_*n*_	Anode dissolution rate
i	Current density
$N_{H_{2}}$	The molar mass of hydrogen
F	Faraday’s constant
*κ*	Conductivity of the electrolyte
*β*	The void fraction of gas bubbles
*T*	Electrolyte temperature
*m*	The correction coefficient of bubble fraction on conductivity
*η* *ω*	Temperature-dependent gradient
Δ*t*	Time intervals
*U*	Machining voltage
Δd	Material removal depth
Δ	Machining gap
$V_{f}$	Feed rate of cathode tool
*t*	Time
*R*_a_	Surface roughness
$P_{in}$	Electrolyte inlet pressure
$P_{out}$	Electrolyte outlet pressure
subscript 0	Initial state
subscript e	Final state
